# The corticotropin-releasing factor-like diuretic hormone 44 (DH_44_) and kinin neuropeptides modulate desiccation and starvation tolerance in *Drosophila melanogaster*

**DOI:** 10.1016/j.peptides.2016.02.004

**Published:** 2016-06

**Authors:** Elizabeth Cannell, Anthony J. Dornan, Kenneth A. Halberg, Selim Terhzaz, Julian A.T. Dow, Shireen-A. Davies

**Affiliations:** a Institute of Molecular, Cell and Systems Biology, College of Medical, Veterinary and Life Sciences, University of Glasgow, University Avenue, Glasgow G12 8QQ, UK; b Section of Cell- and Neurobiology, Department of Biology, University of Copenhagen, Universitetsparken 15, DK-2100 Copenhagen, Denmark

**Keywords:** DH_44_, Kinin, Desiccation, Starvation, Neuropeptide receptor, *Drosophila melanogaster*

## Abstract

Malpighian tubules are critical organs for epithelial fluid transport and stress tolerance in insects, and are under neuroendocrine control by multiple neuropeptides secreted by identified neurons. Here, we demonstrate roles for CRF-like diuretic hormone 44 (DH_44_) and *Drosophila melanogaster* kinin (Drome-kinin, DK) in desiccation and starvation tolerance.

Gene expression and labelled DH_44_ ligand binding data, as well as highly selective knockdowns and/or neuronal ablations of DH_44_ in neurons of the pars intercerebralis and DH_44_ receptor (DH_44_-R2) in Malpighian tubule principal cells, indicate that suppression of DH_44_ signalling improves desiccation tolerance of the intact fly.

Drome-kinin receptor, encoded by the leucokinin receptor gene, LKR, is expressed in DH_44_ neurons as well as in stellate cells of the Malpighian tubules. LKR knockdown in DH_44_-expressing neurons reduces Malpighian tubule-specific LKR, suggesting interactions between DH_44_ and LK signalling pathways.

Finally, although a role for DK in desiccation tolerance was not defined, we demonstrate a novel role for Malpighian tubule cell-specific LKR in starvation tolerance. Starvation increases gene expression of epithelial LKR. Also, Malpighian tubule stellate cell-specific knockdown of LKR significantly reduced starvation tolerance, demonstrating a role for neuropeptide signalling during starvation stress.

## Highlights

• CRF-like diuretic hormone 44 (DH_44_) signalling modulates desiccation tolerance in *D. melanogaster*.
• *D. melanogaster* kinin (Drome-kinin, DK) has a novel role in starvation stress tolerance.
• There are functional interactions between DH_44_ and kinin signalling pathways.

## 1 Introduction

Diuretic and anti-diuretic hormones act on the insect excretory system [12] and are produced by neurosecretory cells in the brain and ventral ganglia. They are released into the haemolymph via neurohemal sites, where they activate their G protein-coupled receptors (GPCRs) located in the Malpighian tubules [2]. Several diuretic peptides have been identified and functionally characterized in *Drosophila melanogaster*, including CRF-like (DH_44_) and kinin (Drome-kinin, DK).

DH_44_ peptide is produced by neuroendocrine cells in the brain, specifically in three bilateral pairs of cells in the pars intercerebralis (PI) with axons extending to the retrocerebral complex of the corpus cardiacum [4]. DH_44_ neurons also receive inputs from the circadian-timing system, which is known to project to the PI [9], [24], [33], and the DH_44_ neurons are involved in rhythms of rest and activity in *D. melanogaster* [9]. DH_44_ neurons are also activated in response to nutritive sugars, a response that could underlie a coordinated response by the gut and Malpighian tubules to feeding [20].

DK is localised to both the brain and the ventral nerve cord (VNC) [7]. In adult *Drosophila*, the brain DK neurons are localized in the lateral horn of the procerebrum and in the subesophageal ganglia [17], [37]. In the VNC, DK neurons project to the heart and abdominal body wall [6].

DH_44_ acts through cyclic AMP to stimulate fluid secretion by Malpighian tubules [4], whereas DK increases fluid secretion by elevating intracellular Ca^2+^ levels and altering chloride shunt conductance [5], [44], [51]. DH_44_ acts on DH_44_ Receptor 2 (DH_44_-R2) localized to tubule principal cells. Another DH_44_ receptor DH_44_-R1 [30], is primarily expressed in the adult brain [10].

DK is encoded by the leucokinin (LK) gene (http://flybase.org/reports/FBgn0028418.html) and acts on the DK receptor encoded by the leucokinin receptor gene, *LKR* [44] (http://flybase.org/reports/FBgn0035610.html). LKR is expressed in tubule stellate cells [4], [26], [44] and also in DH_44_-expressing PI neurons [4] and in the adult gonads [44].

Consistent with the role of DH_44_ as a diuretic peptide [4], knockdown of *DH_44_-R2* expression impairs osmotic stress survival [27]. Recently, DH_44_ has also been shown to increase gut contractions and to modulate waste excretion [20].

DK acts as a diuretic hormone in fluid homeostasis [5], [23], [44], [51] and DK signalling modulates desiccation stress tolerance [37]. Persistent inactivation of the LK neurons or ubiquitous knockdown of *LKR* results in bloating caused by increased haemolymph volume, a phenotype that is not recapitulated by neuronal knockdown of *LKR* [14], [37]. Thus, LK influences fluid homeostasis specifically through action on LKR in epithelial tissues. In addition to diuretic roles for DK, meal termination [1] and food intake [37] is also modulated by the DK neurons.

The co-localisation of LKR to the DH_44_ neurons suggests interaction between the two signalling pathways [4] and may represent a coordinated neuronal circuit regulating fluid homeostasis. Interplay and regulation is not unprecedented in terms of insect neuropeptides as synergistic effects on Malpighian tubule fluid secretion have been previously noted among diuretic hormones, for example between DK and calcitonin-like diuretic hormone [13], and multiple neuronal circuits have been identified as key moderators of tubule function [8]. Co-localisation is also observed between a number of other neuropeptides, including the presence of corazonin expression in DH_44_-R1 expression neurons in both adult and larval brains [31].

Here we have assessed the potential roles of DH_44_, DH_44_-R2 and LKR with respect to fluid homeostasis and stress responses. We demonstrate roles for DH_44_ signalling in desiccation stress; LKR in starvation responses; and interactions between DH_44_ and DK signalling pathways.

## 2 Methods

### 2.1 *Drosophila* stocks

*Drosophila* lines were reared on standard *Drosophila* diet at 22 °C, 45–55% relative humidity with a 12:12 h light:dark photoperiod. GAL4-UAS crosses were reared and maintained at 26 °C. Wild-type Canton-S (CS), ‘cantonised’ *white honey* (*w^h^*), UAS-*mCD8:GFP*, UAS-*pStinger2*, UAS-*p35* and UAS-*reaper* fly lines were acquired from Bloomington Stock Center (Bloomington, IN). The DH_44_-GAL4 driver line (BL 39347) was created by the Janelia Farm FlyLight Project Team, which uses a short fragment of genomic DNA to control GAL4 expression [29], [43], while the UAS-*DH_44_* RNAi line (BL 25804) was created by the Transgenic RNAi Project [39]. The UAS-*LKR* RNAi line (105155 KK) and UAS-*DH_44_-R2* line (102292 KK) were acquired from Vienna *Drosophila* Resource RNAi Center. VDRC crosses were controlled using a VDRC control line gifted from Dr. Edward Green. The capaR-GAL4 line [50] and c724-GAL4 lines [48], [50] were generated in-house previously.

### 2.2 Immunocytochemistry

Immunocytochemistry against DH_44_ and LKR was performed as described elsewhere [34]. After anesthetizing flies on ice, brains were dissected from *Drosophila* in Schneider’s medium (Gibco Life Technologies), and then fixed in 4% paraformaldehyde. Brains were washed with PBTA (0.5% Triton X-100, 0.1% Azide in PBS), blocked with 10% normal goat serum (Sigma) in PBTA, and incubated overnight with DH_44_ antibody at a concentration of 1:4000 [4]. Following a second round of washing and blocking, brains were incubated with anti-rabbit Alexa Fluor 546 or 488 (Life Technologies) overnight at a dilution of 1:1000. After washing again, brains were mounted onto slides and analyzed using confocal microscopy. Labelling with LKR antibody was carried out at a dilution of 1:1000 [44].

### 2.3 Fluorescent-tagged DH_44_ peptide labelling

Ligand receptor assays were performed on live Malpighian tubules from 7–10 days old male wild-type flies using a *Drosophila* DH_44_ analogue conjugated to a high quantum yield fluorophore, BODIPY 543 (TMR)-C5-maleimide (DH_44_-F). The specificity and functional efficacy of DH_44_-F was tested with a ligand competition assay, using 10^−5^ M unlabelled DH_44_; and a tubule secretion assay using 10^−7^ M DH_44_-F, respectively, as detailed elsewhere [23]. Tubules were incubated in 1:1 of Schneider’s medium and *Drosophila* saline containing 500 ng/ml DAPI and 10^−7^ M DH_44_-F for 15 min, prior to being mounted on poly-l-lysine coated glass bottom dishes in PBS and then imaged using confocal microscopy using a Zeiss LSM 510 Meta inverted confocal microscope. Fluorescent signal analysis was performed as described previously [23], [40].

### 2.4 RNA isolation, cDNA synthesis and quantitative (Q)-RT-PCR

RNA was isolated from groups of 8 *Drosophila* (whole fly), 10 *Drosophila* bodies, or 20 heads from flies aged 5–10 days old using TRIzol Reagent (Life Technologies) following the manufacturer’s instructions. RNA levels were quantified using a NanoVue Plus spectrophotometer (GE Healthcare Life Sciences) and then samples were DNAse treated using the DNA-free DNA Removal kit (Life Technologies). Samples were quantified again and cDNA was synthesized from 500 ng RNA using SuperScript II RT (Thermo Fisher Scientific), following manufacturer’s instructions. Q-RT-PCR was performed using TaqMan Probe-Based Gene Expression Analysis (Life Technologies) in an ABI StepOnePlus Detection System (Applied Biosystems) using the following primers and probes: Dm02138400_m1 (*DH_44_*), Dm01824019_g1 (*DH_44_-R1*), Dm01793183_g1 (*DH_44_-R2*), Dm01843317_s1 (*LK*) and Dm01840198_m1 (*LKR*). TaqMan primers for *alpha tubulin 84b* was synthesised by Integrated DNA Technologies (forward-*CCTCGAAATCGTAGCTCTACAC*, reverse-*ACCAGCCTGACCAACATG*, probe-*TCACACGCGACAAGGAAAATTCACAGA*) using sequences similar to those published elsewhere [54]. RT-PCR data was analysed by the comparative C_T_ method [46]. Fold change was compared to a normalized control using a two-tailed one-sample *t*-test with a null hypothesis of no change (i.e. fold change of 1) [36], [45]. Fold changes that were each normalized to a third shared sample were compared using a two-tailed two-sample *t*-test. These are reported on figures as *p < 0.05, **p < 0.01, ***p < 0.001, ****p < 0.0001.

### 2.5 Ramsay fluid secretion assay

Fluid secretion assays using *Drosophila* Malpighian tubules were performed as described previously [19]. Malpighian tubules were dissected in Schneider’s medium and transferred to a 9 μl drop of 1:1 of Schneider’s medium and *Drosophila* saline [4]. Baseline secretion was measured every 10 min for 30 min, after which 1 μl of peptide (DH_44_, DK from Genosphere Biotechnologies, Paris, France; or DH_44_-F from Cambridge Peptides, Birmingham, UK, all used at 10^−6^ M) was added to the drop. Stimulated secretion was measured every 10 min for a further 30 min. The percentage change of basal secretion rates were calculated as previously shown [38].

### 2.6 Stress tolerance assays

Desiccation survival assays were carried out on 5–10 day-old male flies at 22 °C, 45–55% relative humidity with a 12:12 h light:dark photoperiod and were performed by placing flies in empty vials and counting surviving flies until mortality reached 100% [32], [50], [53]. Starvation assays were conducted by placing male flies aged 5–10 days in vials with 1% low melting point agar (Roche), and counting surviving flies until mortality reached 100% [28], [53]. All experiments were run in triplicate with at least 30 flies in each run of specified genotype. Survival data were plotted as Kaplan–Meier curves. Statistical comparisons were made using the logrank test, with estimation of variance (SE) calculated using the Greenwood formula [15]. Hazard ratios were calculated using the Mantel Haenszel approach, as this test has been found to perform more accurately than the log-rank calculation of hazard when using large sample sizes [3]. Where hazard ratio is calculated against two control lines, the more conservative estimate is reported (i.e. closer to 1).

## 3 Results

### 3.1 Desiccation exposure suppresses non-neural DH_44_-R2 expression while starvation increases non-neural LKR and DH_44_ expression

**Fig. 1:**
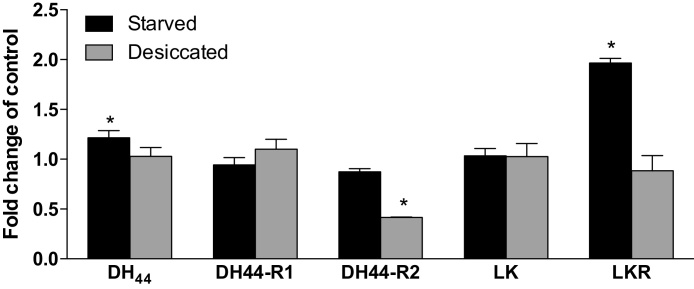
Desiccation and starvation stress impact *DH_44_*, *DH_44_-R2* and *LKR* expression. Quantitative RT-PCR analysis of RNA extracted from whole fly (*DH_44_, DH_44_-R1, LK)* or bodies (*DH_44_-R2, LKR)* of CS *Drosophila* exposed to 24 h of desiccation, 24 h of starvation, or no treatment. Data show no impact of either treatment on *DH_44_-R1* or *LK* expression, but a 60% decrease in *DH_44_-R2* expression following desiccation, and increases in *DH_44_* (22%) and *LKR* (97%) expression following starvation.

Given that LKR is expressed in DH_44_ neurons, and that both DK and DH_44_ are diuretic peptides, putative roles for DH_44_ and DK signalling in desiccation stress were explored by measuring gene expression of *DH_44_*, the DK gene (*LK*) and brain-specific *DH_44_*-R1 in wild type flies, and non-neural *LKR* and *DH_44_-R2* in bodies of wild-type flies, after exposure to 24 h of desiccation, or 24 h of starvation, and compared to a non-stressed control groups. Neither desiccation nor starvation had a significant effect on *DH_44_-R1* or *LK* expression, while *DH_44_-R2* expression was found to decrease significantly (*p* < 0.0001) following desiccation stress, and both *DH*_44_ (*p* *<* 0.05) and *LKR* (*p* < 0.0001) expression increased significantly following starvation stress (Fig. 1).

**Fig. 2:**
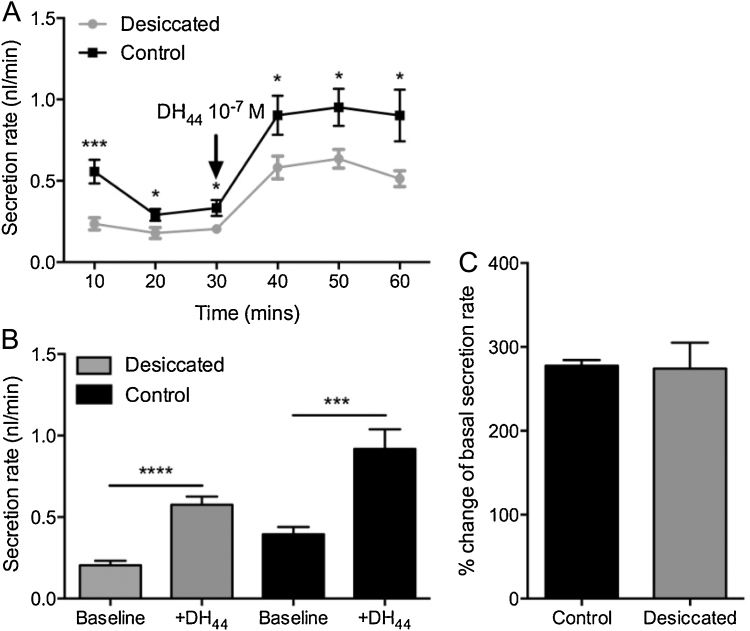
Desiccation stress impacts fluid secretion rate of Malpighian tubules. A, B. Baseline and DH_44_-stimluated secretion rates are significantly lower in desiccated wild-type flies compared to untreated controls. C. The percentage change in secretion rate following stimulation with 10^−7^ M DH_44_ peptide is similar in desiccated wild type flies and untreated controls.

The impact of desiccation on Malpighian tubule function was assessed using a secretion assay. The baseline and DH_44_-stimulated secretion rates of *Drosophila* exposed to 24 h of desiccation are significantly lower than that of control flies (Fig. 2A, B). However, the percentage change in secretion rate following stimulation with DH_44_ peptide is similar in tubules from both desiccated and non-desiccated flies (Fig. 2C).

**Fig. 3:**
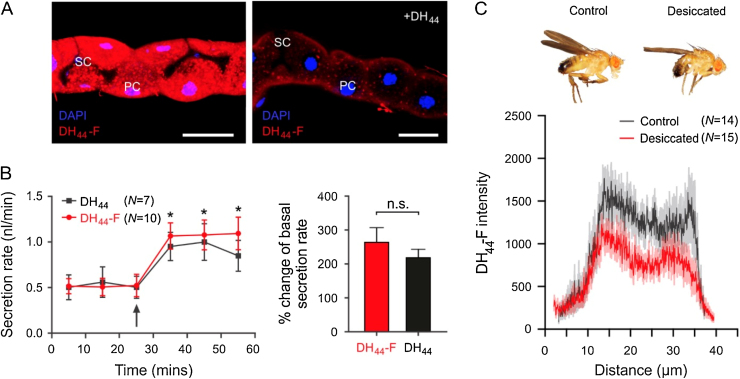
DH_44_ binding to DH_44_-R2 in Malpighian tubules is reduced following desiccation exposure. A. Unlabelled DH_44_ (10^−5^ M) displaces bound fluorescent-labelled DH_44_ (DH_44_-F; 10^−7^ M). B. Both DH_44_-F and DH_44_ significantly increase fluid secretion rate to a similar extent when applied to excised Malpighian tubules. C. DH_44_-F label intensity is reduced in Malpighian tubules of desiccated wild-type flies when compared to unstressed controls.

Potential changes in *DH_44_-R2* receptor abundance following desiccation exposure were assessed using fluorescently labelled DH_44_ peptide (DH_44_-F) binding to intact tubules. The specificity of DH_44_-F binding to tubule *DH_44_-R2* receptors was verified by a ligand competition assay in which unlabelled peptide was able to displace DH_44_-F labelling (Fig. 3A) and by the ability of DH_44_-F to stimulate fluid secretion to a similar extent as unlabelled peptide during secretion assay (Fig. 3B). The intensity of fluorescent signal from DH_44_-F labelling of tubules from desiccated flies was found to be lower than that of the signal from unstressed controls (Fig. 3C).

### 3.2 Manipulations of the DH_44_ neurons indicate a role for DH_44_ signalling in desiccation tolerance

As the data on desiccation-stressed wild-type flies indicated a role for the DH_44_ signalling pathway during desiccation exposure, manipulations of the DH_44_ neurons were performed and their impact on desiccation stress survival was assessed. In order to probe the function of these neurons, a DH_44_-GAL4 line in which GAL4 is expressed under the control of a known short fragment of genomic DNA containing the promoter sequence of the *DH_44_* gene [29] was selected.

**Fig. 4:**
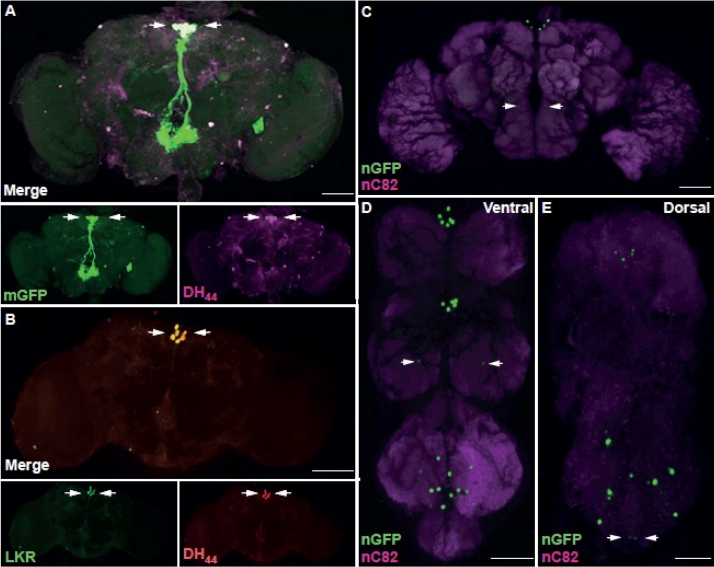
Characterisation of DH_44_ expression pattern in 5–7 days adult CNS. A. Co-expression of *UAS-membrane-bound CD8:GFP* (mGFP) driven by DH_44_-GAL4 and DH_44_ antibody in the adult brain. Co-localisation in the soma of 6 neurons of the pars intercerebralis indicated (arrows). B. Co-expression of LKR and DH_44_ in the adult brain. Co-localisation in the soma of 6 neurons of the pars intercerebralis indicated (arrows). C. *UAS-pStingerII nuclear GFP* (nGFP) driven by DH_44_-GAL4 in the adult brain. Two bilateral clusters of ∼2 smaller neurons in the suboseophageal ganglion indicated (arrows). D. *UAS-pStingerII nuclear GFP* (nGFP) driven by DH_44_-GAL4 in the adult ventral nerve cord (VNC), ventral view. Expression apparent in clusters in the prothoracic, mesothoracic and abdominal (Abg) ganglia. Pair of smaller neurons in the metathoracic ganglion indicated (arrows). E. *UAS-pStingerII nuclear GFP* (nGFP) driven by DH_44_-GAL4 in the adult ventral nerve cord (VNC), dorsal view. Pair of smaller neurons in the distal Abg indicated (arrows). Neuropil counterstained with anti-nC82 (nC82, magenta) where indicated. All patterns of expression are representative of both males and females. All views ventral unless otherwise indicated. Scale bars = 50 μm. (For interpretation of the references to colour in this figure legend, the reader is referred to the web version of this article.)

DH_44_ expression has previously been observed in a restricted number of neurons within the CNS, most notably in two bilateral clusters of 3 neurons localized to the pars intercerebralis (PI) [4], [9], [20], [35], [42]. The DH_44_-GAL4 transgene’s ability to reiterate endogenous gene expression was validated by co-expression with a DH_44_ antibody localizing to the DH_44_ neurons [4]. Expression of the GAL4-responsive mCD8:GFP (membrane-bound GFP) reporter in conjunction with the DH_44_ antibody demonstrated absolute co-localisation in the 6 DH_44_ neurons of the PI (Fig. 4A). In addition we were able to demonstrate, via co-expression of DH_44_ and DK receptor (LKR) antibodies, that these 6 neurons (Fig. 4B), are also positive for LKR expression.

We performed a spatio-temporal assay of *DH_44_* expression within the CNS using the DH_44_-GAL4 transgene driving nuclear (nGFP) as well as membrane-bound GFP (mGFP). In the adult *DH_44_* expression is most notable in the two bilateral clusters of 3 neurons localized to the PI, with these clusters sending characteristic ipsilateral projections through the superior protocerebrum around the oesophageal foramen to form large dendritic arborisations on the prow and flange of the suboesophageal ganglion (Fig. 4C and Supplemental Fig. 1D). These arborisations obscure a further pair of bilateral clusters of smaller neurons that then send projections from the prow (Fig. 4C and Supplemental Fig. 1F), that have been identified as ramifying on the crop and midgut, and are associated with the detection and consumption of nutritive sugars [20].

In the adult VNC, expression is restricted to two sets of bilateral clusters of 3 neurons in the prothoracic and mesothoracic ganglions, an individual pair of smaller neurons in the metathoracic ganglion and finally a large grouping of interconnected neurons in the abdominal ganglion (Abg) (Fig. 4D, E and Supplemental Fig. 1E and G). The neurons in the metathoracic and abdominal ganglion appear to form an interconnected network of dendrites as well as projecting axons to peripheral (non-CNS) structures (Supplemental Fig. 1E and G). Again the most distal dendritic arborisation on the Abg occludes two smaller neurons that send axonal projections to the internal genitalia (Fig. 4E and Supplemental Fig. 1E and G), which, in the female, have been identified as modulating sperm-ejection and storage [35]. It has been shown previously that *DH_44_* expression occurs in the embryo as well as the larva [4], [22], [57], we expanded this to show that expression is also present, though at reduced levels, in L1/L2 stages (data not shown) becoming overt by stage L3, and continuing on in an expanded pattern of expression in the pupal brain and VNC (Supplemental Fig. 1A–C). The more restricted numbers of neurons expressing *DH_44_* in the adult, as compared to the larval and pupal, CNS is most likely a result of neuronal sculpting during metamorphosis, as expression of the GAL4 responsive anti-apoptotic transgene UAS-p35 [25] results in an expanded number of *DH_44_*-positive neurons in the CNS, most notably in the brain (Supplemental Fig. 1H).

**Fig. 5:**
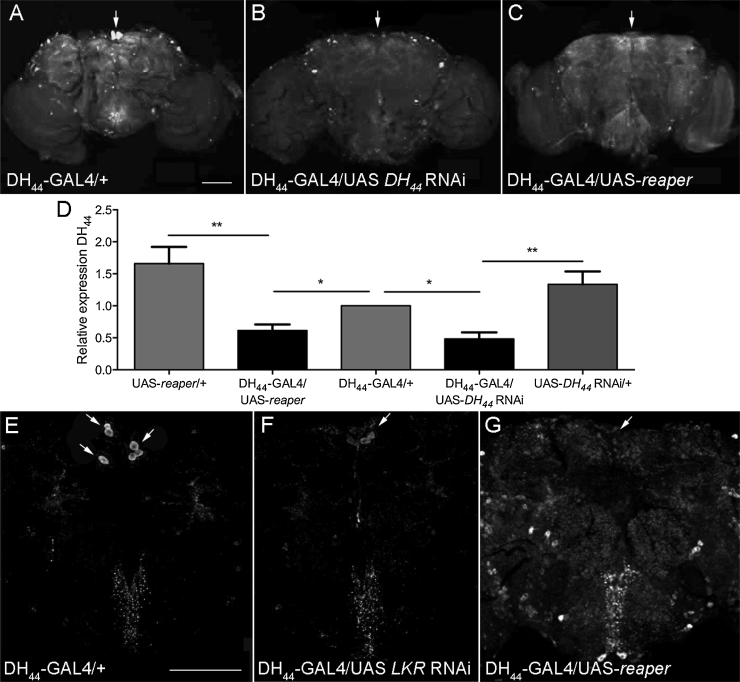
A–C. Elimination of DH_44_ peptide in pars intercerebralis achieved via RNAi knockdown and neuronal ablation. A. Brains from control DH_44_-GAL4/+ progeny stained for DH_44_ show clear labelling in the pars intercerebralis (arrowed). B. DH_44_ staining in the pars intercerebralis is abolished in progeny from cross between DH_44_-GAL4 and UAS-DH_44_ RNAi (arrowed). C. Ablation of DH_44_ neurons via cross between DH_44_-GAL4 and UAS-reaper eliminates the distinctive DH_44_ staining pattern of six neurons in the pars intercerebralis (arrowed). D. Knockdown of DH_44_ gene expression in head upon either DH_44_ neuronal ablation or RNAi knockdown of DH_44_. E–G. Reduction or elimination of LKR expression in pars intercerebralis achieved via RNAi knockdown or neuronal ablation, respectively. E. Brains from control DH_44_-GAL4/+ progeny stained for LKR show clear labelling in the pars intercerebralis (arrowed). F. Decreased intensity of LKR staining in the pars intercerebralis in progeny from cross between DH_44_-GAL4 and UAS-LKR RNAi (arrowed). G. Ablation of DH_44_ neurons in progeny of cross between DH_44_-GAL4 and UAS-reaper eliminates LKR staining in the pars intercerebralis (arrowed).

A targeted RNAi knockdown approach was then used to test whether either DH_44_ or LKR within DH_44_ neurons modulates desiccation tolerance, starvation tolerance, or both. In order to reduce expression of *DH_44_,* DH_44_-GAL4 flies were crossed to a UAS-*DH_44_* RNAi line. Immunochemistry using antibody against DH_44_ peptide showed a total loss of DH_44_ peptide in DH_44_-GAL4/UAS-*DH_44_* RNAi progeny (Fig. 5A, B). Confirmation by Q-RT-PCR showed that *DH_44_* mRNA expression in heads was reduced to approximately 42% of the levels found in parental controls crossed to *w^h^* (Fig. 5D). Immunostaining *LKR* knockdown in the DH_44_ neurons (65% decrease of LKR mRNA levels, data not shown) is also observed (Fig. 5E, F),

Furthermore, in order to probe potential roles for DH_44_ neurons in desiccation and starvation tolerance, genetic ablation of DH_44_ neurons via GAL4-mediated expression of the *reaper* (apoptotic) transgene [55], [56] was performed, resulting in the complete loss of the DH_44_ neurons in the PI as demonstrated by absence of DH_44_ immunoreactivity (Fig. 5C and G) and reduction in overall gene expression (Fig. 5D).

**Fig. 6:**
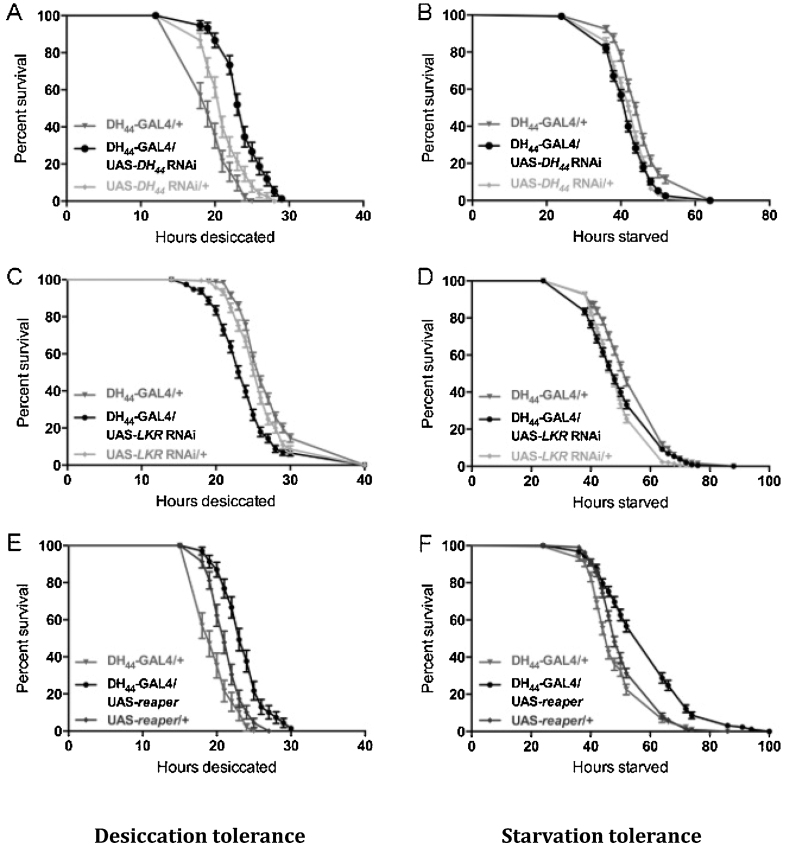
Consequence of targeted *DH_44_* RNAi, *LKR* RNAi and *reaper* in the DH_44_ neurons on desiccation stress (left) and starvation stress (right). A. RNAi knockdown of *DH_44_* in the DH_44_ neurons increases survival time during desiccation stress exposure (*p* < 0.0001). B. RNAi knockdown of *DH_44_* in the DH_44_ neurons did not significantly impact survival time during starvation stress exposure relative to both controls. C. Partial RNAi knockdown of LKR in the DH_44_ neurons resulted in decreased survival time during desiccation stress (*p* < 0.0001). D. Partial RNAi knockdown of *LKR* in the DH_44_ neurons did not significantly affect survival time during starvation stress. E. Ablation of DH_44_ neurons via targeted expression of *reaper* increased survival time during desiccation exposure (*p* < 0.0001). F. Ablation of DH_44_ neurons via targeted expression of *reaper* increased survival time during starvation exposure (*p* < 0.0001).

DH_44_-GAL4/UAS-*DH_44_* RNAi progeny were assayed for desiccation and starvation survival; the latter also controlled for any potential starvation effects during the desiccation stress experiments (Fig. 6). Knockdown of *DH_44_* expression in the DH_44_ neurons was found to significantly extend survival time during desiccation exposure (*p* < 0.0001 against both controls; Logrank test; Fig. 6A). RNAi knockdown of DH_44_ peptide in DH_44_ neurons was associated with at least half the rate of death relative to control groups during desiccation stress (hazard ratio: 0.37, 95% confidence interval [CI]: 0.25–0.54) and an approximately 20% increase in median survival time. Survival time during starvation was not significantly impacted by *DH_44_* knockdown when compared to both parental controls (Fig. 6B). Gravimetric analysis [5] to calculate water content [21] of the DH_44_-GAL4/UAS-*DH_44_* RNAi flies and parental controls showed no significant difference in total body water content between GAL4/UAS-*DH_44_* RNAi flies and parental controls for males and females (Supplementary Fig. 2). Thus, increased desiccation tolerance of DH_44_-GAL4/UAS-*DH_44_* RNAi flies is not due to increased body water retention.

Partial knockdown of *LKR* in the DH_44_ neurons was found to have a different effect compared to knockdown of DH_44_ in the DH_44_ neurons. DH_44_-GAL4/UAS-*LKR* RNAi progeny exhibited significantly reduced survival time during desiccation exposure (*p* < 0.0001 against both controls; Logrank test), with a hazard ratio of 1.75 (95% CI: 1.40–2.18) and an 8% decrease in median survival time (Fig. 6C). Survival during starvation was not significantly impacted by the manipulation of *LKR* expression in DH_44_ neurons when compared to both parental controls (Fig. 6D).

Although knockdown of each *DH_44_* and *LKR* expression in the DH_44_ neurons did not affect starvation tolerance, ablation of the DH_44_ neurons in DH_44_-GAL4/UAS-*reaper* progeny was found to significantly increase survival time during both desiccation stress exposure (*p* < 0.0001 against both controls; Logrank test) (Fig. 6E) and starvation exposure (*p* < 0.0001 against both controls; Logrank test) (Fig. 6F). Ablation of the DH_44_ neurons was associated with less than half the rate of death of controls during desiccation stress (Hazard ratio: 0.38; 95% CI: 0.25 to 0.58) and an approximately 16% increase in median survival time. During starvation stress, ablation of DH_44_ neurons resulted in a hazard ratio of 0.48 relative to parental controls (95% CI: 0.39–0.58) and an increase in median survival time of approximately 18%.

### 3.3 Malpighian tubule response to DH_44_ peptide is not affected by manipulation of DH_44_-producing neurons, although expression of DH_44_-R2 and LKR is altered

One way in which knockdown of DH_44_ in the DH_44_ neurons could potentially influence desiccation tolerance is by altering the abundance or functionality of the DH_44_ receptor, DH_44_-R2 in the Malpighian tubules [27]. As DH_44_-R2 invokes a diuretic effect, compromising its function could potentially promote fluid retention as is observed with the capa neuropeptide receptor, capaR [50]. In order to test this, basal and DH_44_-stimulated fluid secretion rates [4] were measured in intact tubules from flies in which the DH_44_ peptide was knocked down in the DH_44_ neurons and in flies with ablated DH_44_ neurons.

**Fig. 7:**
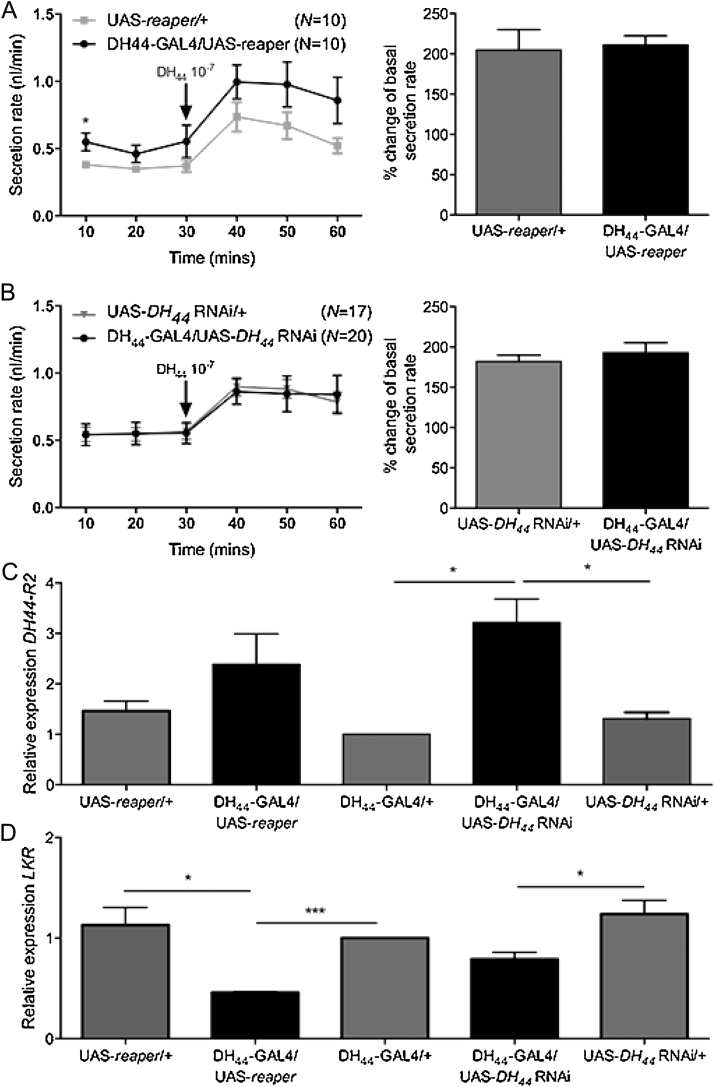
DH_44_ neuron manipulation impacts mRNA expression of DH_44_-R2 and LKR in the Malpighian tubules, but not secretion response to DH_44_ peptide. A. Baseline and DH_44_-stimulated secretion rates are not significantly different between flies with ablated DH_44_ neurons and parental controls. B. Baseline and DH_44_-stimulated secretion rates are similar between DH_44_ knockdown flies and parental controls. C. DH_44_-R2 expression in the Malpighian tubules is increased by RNAi knockdown of DH_44_ in DH_44_ neurons. D. LKR expression in the Malpighian tubules is decreased by ablation of the DH_44_ neurons (* = *p* < 0.05).

In the DH_44_-GAL4/UAS-*reaper* progeny tubules, both baseline secretion and stimulated secretion rates were similar to those of the control progeny, and the percentage change in the stimulated fluid transport rate compared to mean baseline secretion did not differ significantly between the groups (Fig. 7A). Similarly, knockdown of *DH_44_* in the DH_44_ neurons using RNAi did not impact the baseline secretion rate of the tubules or the ability of the tubules to respond to DH_44_ stimulation (Fig. 7B). These results indicate that DH_44_-R2 remains functional in both DH_44_-GAL4/UAS-*reaper* and DH_44_-GAL4/UAS-*DH_44_* RNAi tubules, and that the manipulation of the DH_44_ neurons does not have a feedback effect on DH_44_-R2 function in the Malpighian tubules.

However, changes in mRNA expression of *DH_44_-R2* and *LKR* were observed in Malpighian tubules of DH_44_-GAL4/UAS-*reaper* and DH_44_-GAL4/UAS-*DH_44_* RNAi progeny. *DH_44_-R2* mRNA expression was increased 2.5-fold in tubules of DH_44_-GAL4/UAS-*DH_44_* RNAi flies compared to controls (Fig. 7C). *DH_44_-R2* expression was also higher in tubules of DH_44_-GAL4/UAS-*reaper* progeny, but the difference was not statistically significant. By contrast, *LKR* mRNA expression was decreased by 2.2-fold in tubules of DH_44_-GAL4/UAS-*reaper* flies compared to controls (Fig. 7D). *LKR* expression was also decreased in DH_44_-GAL4/UAS-*DH*_44_ RNAi cross progeny tubules, although this was only significant relative to one parental control.

### 3.4 Knockdown of LKR and DH_44_-R2 in Malpighian tubules impacts fluid secretion, desiccation and starvation tolerance

Having demonstrated the impact of manipulation of neuronal DH_44_ signalling on desiccation survival (Fig. 6) but without effect on Malpighian tubule fluid secretion rates (Fig. 7), putative roles of Malpighian tubule DH_44_-R2 and LKR in desiccation tolerance were assessed by selective RNAi knockdown in either tubule principal or stellate cells, respectively. This was achieved using GAL4 drivers targeted to Malpighian tubule principal (capaR-GAL4) or stellate (c724-GAL4) cells.

**Fig. 8:**
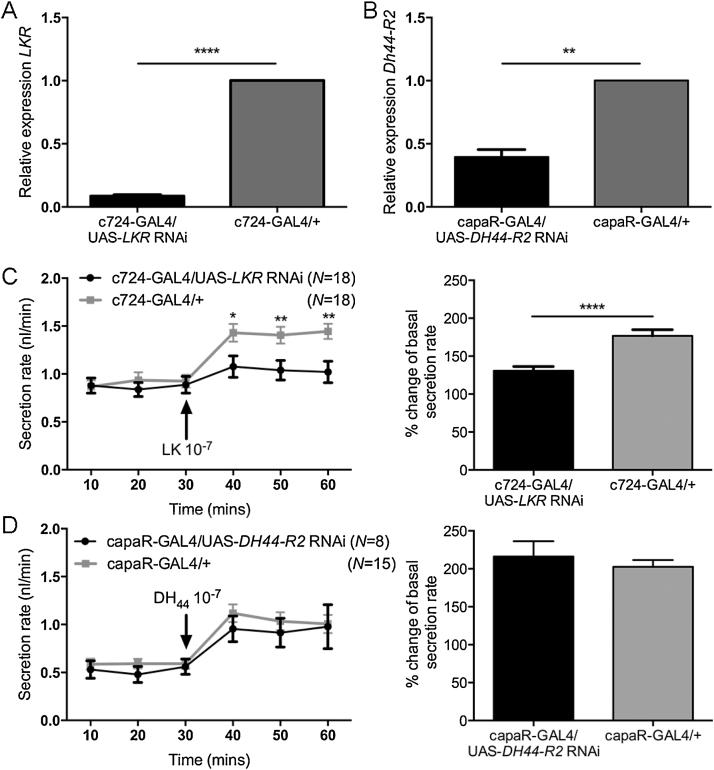
Knockdown of *LKR* in stellate cells of the Malpighian tubules suppresses response of tubules to DK peptide. A. Expression of UAS-*LKR* RNAi in stellate cells of Malpighian tubules results in 91% knockdown of *LKR* mRNA levels in tubules. B. Expression of UAS-*DH_44_-R2* RNAi in principal cells results in 60% knockdown of *DH_44_-R2* mRNA levels in tubules. C. Knockdown of *LKR* in Malpighian tubule stellate cells impairs tubule response to 10^−7^ M DK. D. Knockdown of *DH_44_-R2* in principal cells does not impact basal secretion rate or secretion rate in response to 10^−7^ M DH_44_.

c724-GAL4/UAS-*LKR* RNAi tubules were found to have a 91% reduction in *LKR* mRNA levels (Fig. 8A) compared to parental controls. A 60% reduction in tubule *DH_44_-R2* mRNA levels in capaR-GAL4/UAS-*DH_44_-R2* RNAi flies (Fig. 8B).

The impact of reduced *LKR* and *DH_44_-R2* expression on Malpighian tubule fluid secretion response to either DK or DH_44_, respectively, was assessed by secretion assay. c724-GAL4/UAS-*LKR* RNAi tubules were found to have a similar basal rate as parental controls, but a significantly reduced DK-stimulated secretion rate (Fig. 8C). By contrast, although *DH_44_-R2* was also significantly reduced by targeted *DH_44_-RNAi,* the basal and DH_44_-stimulated secretion rates of capaR-GAL4/UAS-*DH_44_-R2* RNAi tubules were similar to that of the parental control (Fig. 8D). It is likely that the 60% reduction in capaR-GAL4/UAS-*DH_44_-R2* RNAi tubules is still sufficient for significant expression of DH_44_*-*R2. Efforts to obtain a more efficient RNAi knockdown via incorporation of *dicer* did not further reduce *DH_44_-R2* gene expression (data not shown).

**Fig. 9:**
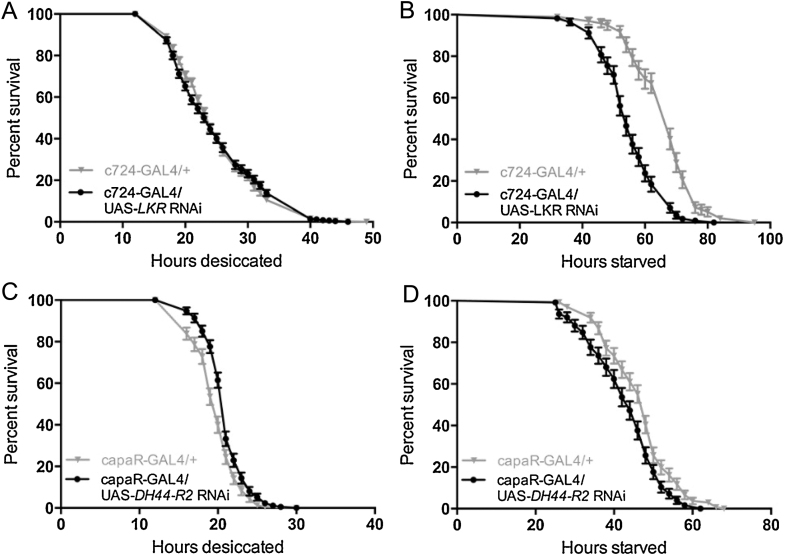
Malpighian tubule diuretic receptors LKR and DH_44_-R2 are involved in desiccation and starvation survival. A. Knockdown of LKR in tubule stellate cells does not significantly impact desiccation tolerance. B. Knockdown of LKR in tubule stellate cells significantly impairs survival during starvation stress (*p* < 0.0001), with a 26% decrease in median survival time. C. Knockdown of DH_44_-R2 in tubule principal cells significantly enhances desiccation tolerance (*p* < 0.001), with a 5% increase in median survival. D. Knockdown of DH_44_-R2 in tubule principal cells significantly impairs survival during starvation stress (*p* < 0.001), with a 9% decrease in median survival.

Having established tubule cell-specific *LKR* and *DH_44_-R2* gene knockdowns, the role of Malpighian tubule LKR and DH_44_-R2 in desiccation and starvation survival was assessed by exposing c724-GAL4/UAS-*LKR* RNAi and capaR-GAL4/UAS-*DH_44_-R2* RNAi flies to stress tolerance assays. Knockdown of *LKR* in tubule stellate cells did not significantly impact desiccation tolerance (Fig. 9A), but significantly impaired survival during starvation (Fig. 9B), resulting in a 3.7 fold rate of death relative to control (95% CI: 2.6–5.2) with a 26% lower median survival time. Inhibition of DK signalling pathways has previously been shown to result in a bloating phenotype of the abdomen [37] and an inflated crop in the gut [1]. The phenotype observed by Liu et al. is thought to be due to an increase in hemolymph volume, potentially due to the loss of DK diuretic action on the Malpighian tubule. Thus, it was expected that *LKR* knockdown in the Malpighian tubules may cause fluid retention that could be detected by gravimetric analysis of body water [21]. However, no difference in water content of c724-GAL4/UAS-*LKR* RNAi compared to parental controls was found. Also, unlike previous studies, these flies did not have a bloated phenotype. However, it may be that targeted LKR knockdown in only tubule stellate cells is not sufficient to impact fluid homeostasis, under conditions of normal LK secretion.

Knockdown of *DH*_44_*-R2* in tubule principal cells significantly improved desiccation survival (Fig. 9C), resulting in a 0.6 fold rate of death (95% CI: 0.48–0.82) and a 5% increase in median survival time. *DH_44_-R2* knockdown significantly impaired starvation tolerance (Fig. 9D), with a hazard ratio of 1.6 (95% CI: 1.2–2.1) and a 9% lower median survival time.

## 4 Discussion

We demonstrate that suppressing the DH_44_ signalling pathways, either by manipulating the DH_44_ neurons or by impacting the DH_44_-R2 in the tubules, improved desiccation survival. We show reduced *DH_44_-R2* transcript levels and concomitantly reduced fluorescent-labelled DH_44_ binding in tubule principal cells upon desiccation. Targeted knockdown of *DH_44_-R2* to Malpighian tubule principal cells also results in improved desiccation tolerance, which may also be modulated by re-absorption by the hindgut. Furthermore, ablation of *DH_44_* neurons or *DH_44_* knockdown in only *DH_44_* neurons both have the effect of improving survival of flies during desiccation stress, possibly due to increased haemolymph volume. However, increased fluid retention was not detected in DH_44_-GAL4/UAS-*DH_44_* RNAi flies.

Our data also imply a role for DH_44_ signalling in starvation tolerance. This is not unprecedented as the DH_44_ neurons also contain LKR, which is involved in feeding regulation [32]. Indeed, ablation of the DH_44_ neurons resulted in increased survival during starvation exposure, while knockdown of *DH_44_* expression in the DH_44_ neurons via RNAi did not clearly impact starvation tolerance. However, in spite of the apparent lack of involvement of *DH_44_* in the DH_44_ neurons in starvation tolerance, a decrease in survival during starvation exposure was observed following knockdown of *DH_44_-R2* in Malpighian tubule principal cells. Consistent with these data was the finding that *DH_44_* gene expression is increased after mild starvation exposure. Impairment of starvation survival by *DH_44_-R2* knockdown could potentially be underpinned by a reduction in food consumption due to bloating, although tubule secretion rates in *DH_44_-R2* knockdown flies are similar to control flies, and no bloating of these flies was observed.

The involvement of the DH_44_ neurons in starvation, however, is clearly indicated by the finding that ablation of these neurons greatly improves starvation survival. These neurons may be involved in circuitry that coordinates the physiological response to starvation, a finding that is perhaps consistent with the involvement of these neurons in nutrient sensing and the co-localization of LKR in these neurons, which may impact feeding behaviour [1], [20].

As with the DH_44_ peptide, a role for the diuretic hormone DK in desiccation tolerance can be hypothesized based on the finding that other diuretic hormones impact desiccation survival in *Drosophila* [32], [47], [50], [53]. Surprisingly, evidence for the involvement of DK signalling in desiccation tolerance from this study was limited. No changes in either whole fly *LK* expression or non-neural *LKR* expression (i.e. body samples) were found following 24 h of desiccation exposure. Consistent with these results was the finding that knockdown of *LKR* in the stellate cells of the Malpighian tubules does not impact desiccation survival. However, it may be that compensatory mechanisms occur via other neuropeptides which act through principal cells e.g. capa, DH_31_ and DH_44_, to maintain fluid secretion rates in stellate-cell *LKR* knockdown flies. Intriguingly, knockdown of *LKR* in DH_44_ neurons reduced desiccation survival. Also, manipulation of DH_44_ levels in the DH_44_ neurons via neuronal ablation or DH_44_ knockdown resulted in significantly reduced expression of the tubule-specific *LKR*. Thus, the DH_44_ and DK pathways interact, and could be co-regulated. Interactions between different neuropeptides and even classical neurotransmitters in the form of modulatory circuits have been proposed to occur elsewhere in the *Drosophila* brain [8], [49].

DK has demonstrated roles in feeding behaviour [1], [37], so a role in starvation tolerance is also plausible. Ablation of DH_44_ neurons (resulting in lack of neuronal LKR), but not RNAi knockdown of *DH_44_* resulted in increased tolerance to starvation survival. By contrast, tubule stellate-cell specific knockdown of *LKR* results in reduced starvation survival; and expression of non-neuronal *LKR* is significantly increased under starvation conditions. These novel findings may be explained by the complex role of the Malpighian tubules, beyond osmoregulation. The Malpighian tubules are critical tissues not only for fluid homeostasis, but also for detoxification [11], [16], [18], [52]. Evidence indicates that lipid metabolism in the fat body is a particularly crucial source of energy during starvation [41]. Lipid mobilisation results in waste products being released into the hemolymph, which are then taken up by the Malpighian tubules for processing and excretion [41]. Interference with this process by reducing the ability of the Malpighian tubules to increase fluid secretion, potentially in response to changes in hemolymph osmolarity, could impact on the ability of the organism to mobilise energy resources. Thus, it could be interference with the role of the Malpighian tubule in detoxification, rather than in fluid homeostasis, that impacts starvation tolerance when *LKR* expression is reduced in the tubules. Moreover, the *LKR* gene has seven predicted binding sites for transcription factors [44], thereby providing several possible sites that could be used to modify gene expression during stress exposure.

Recently, insect diuretic neuropeptides that act on Malpighian tubules to modulate fluid homeostasis e.g. capa, kinin and DH_44_, have been found to modulate stress tolerance, metabolism and reproduction—and so are critical for organismal survival. The challenge will be to unravel the precise mechanisms of function of these neuropeptides, and to understand environmental ‘cues’ for potential co-regulation of neuropeptide gene expression, release, activation and signalling.

## Author contributions

EC, AJD, KAH and ST performed the experiments, analysed the data and EC, SD, AJD wrote the manuscript. All work was conducted in the laboratory of SD/JATD, who designed experiments in conjunction with ST/EC/AJD/KAH.

## Conflict of interest

The authors declare that they have no conflict of interest.
