# Supplementary material for: The corticotropin-releasing factor-like diuretic hormone 44 (DH44) and kinin neuropeptides modulate desiccation and starvation tolerance in Drosophila melanogaster
Source: Peptides. 2016 Jun;80:96–107. doi: 10.1016/j.peptides.2016.02.004 (PMC4889782; doi:10.1016/j.peptides.2016.02.004)
Supplement: Supplementary file 1 [file mmc1.docx]

**Supplemental Figures**

**
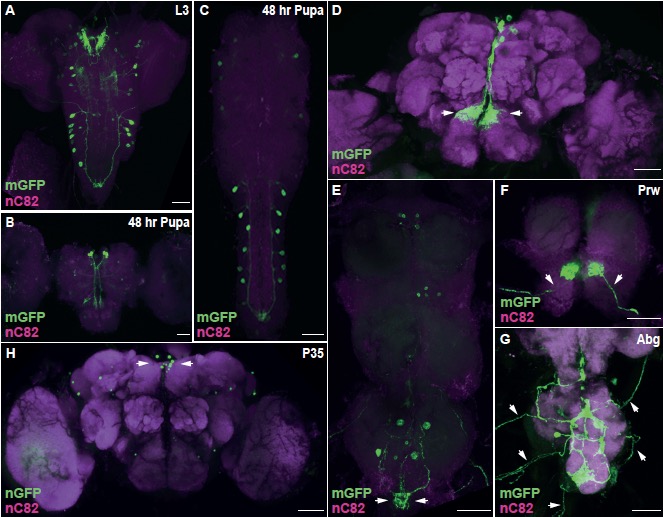
**

**Supplemental Figure 1. Spatio-temporal characterisation of DH_44_ expression in the CNS.**

**A.** DH_44_-GAL4 driving UAS-*mCD8::GFP* (mGFP) in an L3 CNS, exhibiting expanded neuronal expression, with neurons of the developing nerve cord exhibiting an interconnected network of projections. **B.** 48 hr UAS-*mCD8::GFP*; DH_44_-GAL4 brain, exhibiting expression in a cluster of neurons within the PI and individual neurons in the lateral protocerebrum, sending ipsi- and contra-lateral projections to the prow in the sub-oesophageal ganglion (SOG). **C.** 48 hr UAS-*mCD8::GFP*; DH_44_-GAL4 pupal ventral nerve cord (VNC), with neurons exhibiting an interconnected network of projections throughout. **D.** 5 day- old UAS-*mCD8::GFP*; DH_44_-GAL4 adult brain, exhibiting expression in 6 pairs of neurons in the pars intercerebralis (PI) which send axonal projections to the prow (Prw) and flange of the suboesophageal ganglion (SOG). Note the dendritic arborisation at the Prw (arrows) occludes visualisation of two pairs of neurons, which send projections to the crop and midgut. **E.** 5 day old UAS-*mCD8::GFP*; DH_44_-GAL4 adult VNC, exhibiting expression in clusters of neurons within the the pro- and meso-thorocic ganglia and a network of neurons within the abdominal ganglion (Abg). **F.** Detail of the SOG of a 5 day old UAS-*mCD8::GFP*; DH_44_-GAL4 adult, exhibiting expression in axonal projections (arrows) originating from two bilateral pairs of neurones in the prow (Prw). Note the dendritic arborisation (arrows) occludes visualisation of the neuronal bodies. **G.** Detail of the Abg of a 5 day old *UAS-mCD8::GFP*; DH_44_-GAL4 adult, with neurons exhibiting both an interconnected network of dendritic connections and axonal projections (arrows) to peripheral (non-CNS) tissues. **H.** DH_44_-GAL4 driving UAS-*pStinger2* (nGFP) as well as UAS-*p35* (anti-apoptotic transgene; P35) in a 5 day old adult brain, exhibiting expanded expression throughout, notably in the bilateral pair of neuronal clusters in the PI (arrows). Neuropil counterstained with anti-nC82 (nC82, magenta). All patterns of expression are representative of both males and females. All views ventral. Scale bars = 50μm.


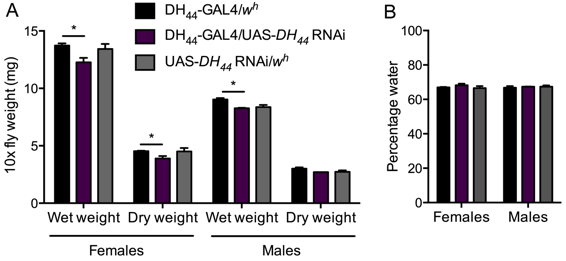


Supplemental Figure 2. Flies with *DH_44_* RNAi knockdown in DH_44_ neurons weigh significantly less than control, but have similar percentage of body water. A. Flies with genotype DH_44_-GAL4/*w^h^* have a significantly higher wet weight than those with genotype DH_44_-GAL4/UAS-*DH_44_* RNAi regardless of sex. Female flies with genotype DH_44_-GAL4/*w^h^* have a significantly higher dry weight than females with genotype DH_44_-GAL4/UAS-*DH_44_* RNAi. *N*=3 for all genotypes. B. Male and female flies with DH_44_ knockdown in the DH_44_ neurons have a percentage body water that is similar to progeny of control crosses. See part A for genotype legend. Percentage body water calculated as in [[21](#_ENREF_21)]. Data show mean ± SEM, **p*<0.05.
